# Supplementary figures and images for: Illuminating the effect of beneficial blue light and ROS-modulating enzymes in Dupuytren’s disease
Source: PLoS One. 2021 Jul 16;16(7):e0253777. doi: 10.1371/journal.pone.0253777 (PMC8284832; doi:10.1371/journal.pone.0253777)

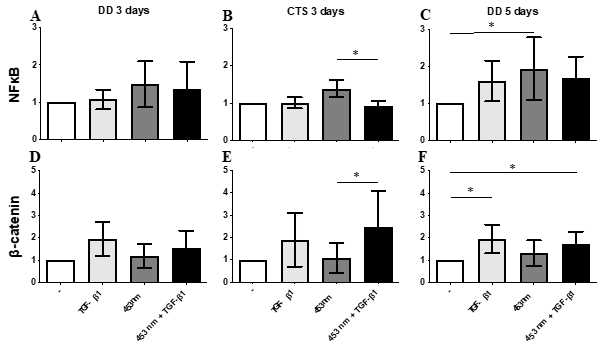

Supplement: S2 Fig — Relative NFκB protein expression (A–C). In irradiated and activated CTS fibroblasts NFκB protein expression was significantly inhibited in comparison to irradiated CTS fibroblasts on day 3 (B). Moreover, the irradiation elevated NFκB protein expression compared to resting fibroblasts, and this effect was significant in DD fibroblasts on day 5 (C). * p ≤ 0.05. Bars represent mean ± SD of individual experiments indicated (n = 7). Relative β-catenin protein expression (D–F). In irradiated and activated CTS fibroblasts, the β-catenin expression was significantly reduced compared to a solely blue light irradiation (E) on day 3. On day 5, β-catenin expression was significantly increased in activated as well as in irradiated and activated DD fibroblasts (F) compared to resting fibroblasts. * p ≤ 0.05. Bars represent mean ± SD of individual experiments indicated (n = 8). (TIF) [file pone.0253777.s002.tif]
