# Supplementary material for: Illuminating the effect of beneficial blue light and ROS-modulating enzymes in Dupuytren’s disease
Source: PLoS One. 2021 Jul 16;16(7):e0253777. doi: 10.1371/journal.pone.0253777 (PMC8284832; doi:10.1371/journal.pone.0253777)
Supplement: Attachment — Submitted filename: Response to Reviewers_R2_final.docx [file pone.0253777.s004.docx]

**Response to Reviewers**

*Review Comments to the Author:*

**Reviewer #1:** (No Response)

**Answer:** No answer required

**Reviewer #2:** I have reviewed the authors’ responses to my comments as well as the highlighted changes in the re-submitted manuscript, and I feel that very little has been changed in their response to my feedback. Specifically:

**1.** No information has been added TO THE MANUSCRIPT regarding my comment about “Please clarify how long on average, it took for fibroblasts to reach confluence, and how long between surgical extraction of tissue samples and blue light irradiation”

**Answer:** The following paragraph has now been added to the manuscript: “After an average time of 14 days after extraction the DD fibroblasts are confluent. After a further 14 days, we start with the experiments, usually in cell culture passage 3-5, in exceptional cases up to cell culture passage 8. The prerequisite for using cell culture passage 8 is that fibroblasts proliferation rate and morphology have not changed.”

**2.** No information has been added TO THE MANUSCRIPT regarding my comment about “How was non-toxicity of TGF-β1 and H_2_O_2_ established?”

**Answer:** The following paragraph was now added to the manuscript: “Previously conducted studies by our working group determined non-toxicity of TGF-β1 and H_2_O_2_ using a CellTiter-Blue Cell Viability Assay from Promega. According to the results, we conclude that the use of TGF-β1 (1 ng, 5 ng, 10 ng/ml) and H_2_O_2_ (10 µM, 20, µM, 50 µM) over the course of 5 days has not toxic effect (S1 Fig). The corresponding figure was inserted in the Supporting information.

**3.** Grammar remains an issue with this paper. Some examples follow (but there are many more which need to be identified and corrected), and I believe that a native English speaking editor would be able to assist with these matters to aid clarity:

**Answer:** Thank you very much. The entire manuscript was checked again for errors of grammar, punctuation and syntax with help of a native English speaking editor. All changes were marked in the Revised Manuscript using the yellow colour.

**4.** Page 6: “Prior delivery the irradiance of the LED-device was proven using an integrating (Ulbricht) sphere.” Do you mean, “Prior to use, the irradiance of the LED-device was verified using an integrating (Ulbricht) sphere.”?

**Answer:** Not quite. What we wanted to state is that, before being released for delivery the irradiance of the LED-device was validated by the manufacturer (Philips Technology Research Laboratories, Aachen, Germany) using an integrating (Ulbricht) sphere. In our laboratory we analysed the exact irradiation time with the corresponding distance with the precise hand radiometer RM 21 (Dr. Gröbel UV-Elektronik GmbH, Ettlingen, Germany). Now this was specified in the manuscript accordingly.

**5.** Page 7: The following sentence remains unclear: “According to the manufacturer’s protocol 25 μl, respectively 12.5 μl supernatant were given in a 96-well plate together with 25 μl, respectively 37.5 μl Assay Buffer.”

**Answer:** The sentences have now been adjusted accordingly: “According to the manufacturer’s protocol 12.5 μl or 25 μl supernatant were given in a 96-well plate and adjusted to a final volume of 50 μl with Assay Buffer. At first 12.5 µl supernatant were used and if the achieved mathematical value was not within the linear range, the experiment was repeated with 50 µl supernatant. Thereafter, 40 μl of the reaction mix (33 μl assay buffer, 3 μl, 40 mM NADPH solution, 2 μl GR solution) were given to the samples.”

**6.** Page 7: “Respectively, 10 μg or rather 20 μg protein were mixed…” So, which was it: 10 μg or 20 μg? Please be accurate.

**Answer:** The information was specified as detailed below: In general, 10 μg of protein or in the case of NOX4, Mn-SOD or NFκB 20 μg of protein were mixed…”

**7.** Page 7: “…anti-Western marker in TBST, who was added for 1 h (RT).” Rather than the use of word “who”, it should probably be the word “that”.

**Answer:** The sentence has been adjusted accordingly.

**8.** Page 8: I see that the following is still in place: “(cf. page 5)”. I have looked at page 5 and I am unable to discern what this means.

**Answer:** The term „standard medium” was now specified on page 5 as detailed below: Briefly, tissue samples were washed twice with phosphate buffered saline (PBS), cut into small pieces (0.2 x 0.2 mm) applied in a petri dish (10 cm) with 13 – 15 ml DMEM ((4.5 g/l glucose, with 𝛼-glutamin (ThermoFisher, Massachusetts, USA)) with 10 % FBS, 1 % penicillin/streptomycin, 1 x sodium pyruvate (as in the following described as standard medium) and incubated at 37 °C by 5 % CO_2_.

**9.** Page 12: Use of the word “erased” is inappropriate both here and in a caption to figure S 1 Fig. Relative NFκB protein expression (A – C). Please replace with a more meaningful word.
Non-scientific language and colloquialisms such as those used on page 13 (as examples) are not appropriate. Please replace: “remarkable” (do you mean that the assumption was prescient, or something to be remarked upon?)

**Answer:** The word “erased” has been replaced with the more appropriate alternative “reduced” in the text and the associated figure caption.

We used the word “remarkable“ because the findings of Taflinski et al. were unexpected and therefore of high interest. We propose writing „…this assumption was noteworthy, because …“

**10.** Starting a sentence with “Anyway” is inappropriate

**Answer:** The sentence has been adjusted accordingly.

**11.** Page 13: “As such an antioxidant isoenzyme, it plays an…” (Delete “it”)

**Answer:** The sentence has been adjusted accordingly.

**12.** Page 13: The sentence beginning with “Whereas Riedl et al. evaluated the positive efficacy of a topical gel containing liposomal encapsulated recombinant…” is not complete and needs to be reviewed.

**Answer:** You are completely right. The senetence was incomplete and was rewritten as follows: “Riedl et al. evaluated the positive efficacy of a topical gel containing liposomal encapsulated recombinant human SOD in the treatment of Peyronie's Disease, a painful fibroproliferative disorder of the penis which is pathophysiologically comparable to DD.”

**13.** Page 14: The following does not make sense: “In TGF-β1-activated DD fibroblasts, the NOX4 expression was hardly affected and the additional irradiation slightly gained NOX4 expression…” Clarify please.

**Answer:** We have revised the sentence as follows:

In TGF-β1-activated DD fibroblasts, the NOX4 expression was hardly affected and the additional irradiation only led to a slight increase of NOX4 expression, which was not significant (Fig 2 E, F).

**14.** Page 14: The following phrase is not clear: “…further accomplishes leads to significant inhibition of…” Please clarify

**Answer:** The sentence has been adjusted accordingly.

**15.** Page 14, Line 332: Replace ‘mayor” with “major”

**Answer:** The word has been adjusted accordingly.

**16.** Page 15, Line 341: Replace “und” with “and”

**Answer:** The word has been adjusted accordingly.

**17.** Page 15, final sentence: Describing something as “rather significant” is not appropriate in a scientific sense. It is either significant or not.

**Answer:** The sentence has been adjusted accordingly.

**18.** Further, one matter that was not obvious to me the first time around: - the temperature of cell culture plates in the experimental groups “never exceeded 38°C” suggests that there was potentially a marked increase in temperature above room temperature that could have affected the results. It also suggests that there was a range of different temperatures across the experimental conditions which may theoretically have affected the results. Firstly, please add to the manuscript what the temperature range was and the mean temperature for each experimental condition. The large differential in temperature with the control plates (kept at 25oC) suggests that this could have been a confounder to the results. Can the authors explain IN THE MANUSCRIPT whether this apparent temperature differential may have had an effect on the outcomes?

**Answer:** We are sorry for causing a misunderstanding here. It was not the case that the irradiated plates were incubated at 38 °C and the control plate at 25 °C for an extended period of time. The experimental procedure was conducted in the following way: The cell culture plates were in general incubated in the humidified atmosphere at 37 °C. For the short irradiation time (16.7 min) irradiated CTS and DD fibroblasts were exposed to room temperature (18 – 21 °C) under the LED device and for the same duration of time control DD and CTS fibroblasts were stored in a *Biometra OV3 Hybridisation* oven at 25°C, in order to have comparable temperatures in both experimental setups (+/- irradiation). This was evaluated in control experiments using a digital thermometer, determining that irradiated DD and CTS fibroblasts never exceeded a temperature of 38 °C and control fibroblasts were kept at a temperature of 37 °C. We specified this procedure in the manuscript. This does not mean that the range of temperature was varying so much. Maybe we better erase the last sentence, if it leads to confusion?
